# The Hospital Movement in the United States

**Published:** 1905-12-16

**Authors:** 


### HOSPITAL ADMINISTRATION.

### CONSTRUCTION AND ECONOMICS.

### THE HOSPITAL MOVEMENT IN THE UNITED STATES.

### THE MASSACHUSETTS STATE HOSPITAL AT TEWKSBURY.

By our Special Commissioner.

(*Continued from page* 169.)

#### II. SPECIAL FEATURES.

General Points of Interest.

*A Capital Account and Annual Valuation.*—It is worthy of note that a capital account is kept in connection with the State Hospital, and that the law requires that an annual inventory shall be taken by an independent professional auditor who appraises the real and personal property of the institution belonging to the Commonwealth and states its total value as certified by him at the end of each financial year. This valuation includes the whole of the real and personal estate and shows the value of the live stock and of the produce on hand at the farm, in the garden, and in each department of the hospital, including the ready-made clothing, the dry goods, the provisions and groceries, the drugs and medicines, the fuel, the carriages and agricultural implements, the machinery and mechanical fixtures, the beds, bedding and other furniture, and the personal property in the superintendent's department. It also divides the real estate into buildings and land, each of which is valued separately. English experience goes to show that a valuation of this kind, and a system of book-keeping which renders the capital account distinct from the ordinary income and expenditure account, makes for economical administration each year. A separate account and valuation is also given of the money set apart for permanent improvements in the institution plant, and the accounts show by separate statements the details of all expenditure of this kind. A strict eye is kept upon the working of the farm, and care is taken to provide that its yield shall as far as possible mark an improvement each year.

*The Training School for Nurses.*—There is an excellent training school for nurses under the superintendence of Miss Rachel Bourke, an English woman who has been educated in America, and whom it was a pleasure to see so happy and enthusiastic in her work. This school has been working for thirteen years. Its course includes thorough hospital training and instruction in mental diseases, therapeutics, materia medica, bacteriology and pathology, under a corps of instructors including members of the medical staff. This school has been accepted amongst those whose graduates are eligible for the New York Registry of Nurses.

*The Medical Staff.*—The active medical and surgical work done at this State Hospital may be gathered from the fact that although there were 5,104 inmates under care during the year 1904, of which 684 were insane patients, the average weekly number of patients was only 1,491, which goes to prove that the State Hospital is serving a useful purpose to hundreds of sick of the pauper class. We were very much interested and pleased by our inspection of this State Hospital, the condition of which reflects the highest credit upon Dr. John H. Nichols, the Superintendent, and the other resident officers, as well as upon the Trustees who are immediately responsible to the public for its administration. We noticed with pleasure, too, that portraits of Dr. Fisher, and of Dr. Howard, were hung in the Board-room and pointed out with pride and gratitude. Each of them was formerly Medical Superintendent of the State Hospital, and to them, with Dr. Nichols, its present high state of efficiency is chiefly due.

All Honour to the Medical Superintendents.

The great progress and development of this institution, compared with others of its type in various States, is in large measure due to the circumstance that, at the time when General Butler swept this Augean stable of nepotism it was fortunate enough to secure the services of Dr. W. E. Fisher as medical superintendent, who now presides so ably over the affairs of the Presbyterian Hospital in New York. Following him came Dr. Howard, the present zealous and indefatigable administrator of the Massachusetts General Hospital, who succeeded Dr. Fisher in the office of medical superintendent. And after them came Dr. John H. Nichols, the present superintendent, whom his trustees describe as "a capable and devoted leader, alert and discreet in the oversight of the hospital and its affairs." After all, despite everything that has been said to the contrary, evidence increases that, providing the managers of a great hospital will take the trouble to secure the services of a really able administrator for the office of medical superintendent, and will give him the entire management and direction of the whole establishment, with the exception of the treatment of the patients, they will so secure progressive development, economical administration, the introduction of novel improvements with discreet regularity, and the highest welfare of the institution and its inmates. There is no other method by which these things can be so certainly done. After the late General Butler, backed by public opinion, though it cost him to his eternal honour, the support of the caucus of those days, supplied the initiative and force to cleanse and reconstitute the administration of this hospital upon a sound and lasting basis, to his successors belongs the credit that the men chosen as medical superintendents since have proved to be amongst the ablest to be found in the United States.

Brains at a Discount.

Our inspections have impressed us, however, with the feeling that, at the present time, the Americans will not and do not pay adequately for brains. This is natural perhaps for the reason that, the majority believe in one-man enterprise which leads them to conclude, half-unconsciously possibly but with precise decision, that if a man has brains he will start out and make a business of his own, so that the product of his brains will so return to him the maximum value in money which they are capable of producing. As an able medical superintendent said to the writer when discussing this question and lamenting the hardship which some men have to suffer in consequence: "I have deliberately refused to go into anything else though I have had wealth placed within my reach if I would do so, for the reason, that I want to give the best years of my life to doing the utmost good I can for others in this work of hospital administration, which has great attractions for me personally on general and scientific grounds." No doubt a few of the abler men, in high position in the United States, recognise that, there are some of their fellow countrymen possessed of the best brains who hold the same views and wish to take a similar course. The sooner those in authority recognise that the United States is producing every day noble men of the type in question, with really great ability, the better will it be for the efficient and economical administration of public institutions and of many other matters of vital importance to the well-being of the American people.

The Branch Hospital for Consumptives.

This establishment is situated about a quarter of a mile from the main buildings, in a pine wood, and faces the south. These buildings were erected several years ago. Their design and the spaciousness of the day-room as well as the fact that the plan includes many of the points to which most modern and authoritative opinion attaches importance, was a pleasant surprise, having regard to the date when the buildings were erected. Nothing could be better, for instance, than the aspect of the buildings, which are so placed as to render it possible for the inmates, in the coldest weather, to sit out in the sunshine every day under the most favourable conditions. It is just to state that no institution yet planned has exhibited a better appreciation of the importance of such provision in designing a sanatorium, nor has succeeded in securing it better.

The complete out-door treatment was attempted in a camp in the pine woods on a small scale. The new departure having proved a success, a new and larger camp was in course of erection at the time of our visit. Altogether 439 cases of tuberculosis were treated in this hospital during the year, of whom twenty-five per cent. at least died. This shows that accommodation of the highest type, admirably suited to be confined as it ought to be confined to the cure of the earlier cases of tuberculosis, and the exclusion of those in the last stage, is being largely misapplied by the admission of advanced cases which are hopeless at the time they enter the buildings. We hope the management may soon classify the cases, making provision for the reception of the advanced cases in a separate building, and confine this really excellent cure-house to those patients only who are in the earlier stages of the disease, where recovery may be hoped to follow continuous treatment. We hope too the plan at present being pursued for the continuous fresh-air treatment of the patients will be extended and made as effective as possible.

